# Clonal relatedness in tumour pairs of breast cancer patients

**DOI:** 10.1186/s13058-018-1022-y

**Published:** 2018-08-09

**Authors:** Jana Biermann, Toshima Z. Parris, Szilárd Nemes, Anna Danielsson, Hanna Engqvist, Elisabeth Werner Rönnerman, Eva Forssell-Aronsson, Anikó Kovács, Per Karlsson, Khalil Helou

**Affiliations:** 1 Department of Oncology, Institute of Clinical Sciences, Sahlgrenska Cancer Center, Sahlgrenska Academy at University of Gothenburg, Box 425, SE-405 30 Gothenburg, Sweden; 2 Swedish Hip Arthroplasty Register, 405 30 Gothenburg, Sweden; 3 Department of Clinical Pathology and Genetics, Sahlgrenska University Hospital, 413 45 Gothenburg, Sweden; 4 Department of Radiation Physics, Institute of Clinical Sciences, Sahlgrenska Cancer Center, Sahlgrenska Academy at University of Gothenburg, 405 30 Gothenburg, Sweden

**Keywords:** Tumour clonality, Bilateral breast cancer, Ipsilateral breast cancer, Intertumour heterogeneity, Similarity index, Multiple breast cancer

## Abstract

**Background:**

Molecular classification of tumour clonality is currently not evaluated in multiple invasive breast carcinomas, despite evidence suggesting common clonal origins. There is no consensus about which type of data (e.g. copy number, mutation, histology) and especially which statistical method is most suitable to distinguish clonal recurrences from independent primary tumours.

**Methods:**

Thirty-seven invasive breast tumour pairs were stratified according to laterality and time interval between the diagnoses of the two tumours. In a multi-omics approach, tumour clonality was analysed by integrating clinical characteristics (*n* = 37), DNA copy number (*n* = 37), DNA methylation (*n* = 8), gene expression microarray (*n* = 7), RNA sequencing (*n* = 3), and SNP genotyping data (*n* = 3). Different statistical methods, e.g. the diagnostic similarity index (SI), were used to classify the tumours as clonally related recurrences or independent primary tumours.

**Results:**

The SI and hierarchical clustering showed similar tendencies and the highest concordance with the other methods. Concordant evidence for tumour clonality was found in 46% (17/37) of patients. Notably, no association was found between the current clinical guidelines and molecular tumour features.

**Conclusions:**

A more accurate classification of clonal relatedness between multiple breast tumours may help to mitigate treatment failure and relapse by integrating tumour-associated molecular features, clinical parameters, and statistical methods. Guidelines need to be defined with exact thresholds to standardise clonality testing in a routine diagnostic setting.

**Electronic supplementary material:**

The online version of this article (10.1186/s13058-018-1022-y) contains supplementary material, which is available to authorized users.

## Background

Approximately 2–15% of women previously diagnosed with breast cancer will develop a second primary carcinoma in the contralateral breast during their lifetime [1, 2]. Interestingly, the risk of developing a breast tumour in the contralateral breast is 2–6-fold higher in breast cancer patients than the risk of developing a first primary breast cancer in the general population [2]. These findings indicate a clonal relationship between bilateral breast cancers as well as a consequence of genetic predisposition and treatment [2, 3]. However, discordance in histologic patterns between bilateral tumours suggests that the majority of bilateral breast cancers have independent tumour origins [4]. Clonality is defined as two tumours deriving from the same progenitor cell that previously underwent malignant changes and gave rise to both of the detected tumours [5]. Consequently, in the early development of the two clones the driver events of the progenitor cell (e.g. copy number alteration (CNA), DNA methylation, mutation, and gene expression profiles) need to have been identical. Due to heterogeneity in subclonal drifts, the variability between the two clones results from the accumulation of diverse molecular changes associated with tumour progression [6]. Nevertheless, similarities in certain tumour features might be due to genetic predisposition and shared environment instead of indicating metastatic spread.

Ipsilateral (unilateral) secondary tumours occur in 10–15% of patients undergoing breast-conserving surgery and radiation therapy [7]. At present, the concordance of hormone receptor status in tumour pairs is the main factor when evaluating potential clonal relatedness of two breast tumours. Clinical characteristics of breast tumours with independent origin are the presence of an in situ component in the second tumour, different degrees of differentiation, different histological subtypes (e.g. invasive carcinoma no special type (NST), invasive lobular carcinoma, tubular, medullary, etc.), absence of locoregional or distant metastases, long time interval between the two tumours, and differences in stage and anatomic location [8, 9]. Determining the concordance of histopathological characteristics between multiple breast carcinomas is insufficient for discerning whether multiple tumours are true recurrences of the primary tumour (clonal) or a new unrelated primary lesion (independent tumour) [10]. Bilateral tumours are currently clinically diagnosed as two different entities, while ipsilateral tumours are classified as local recurrences [1]. Clonal recurrences can represent treatment failure of the first tumour, warranting a change of therapy for the second tumour. Contrastingly, two independent tumours with the same clinical features can be treated similarly since the treatment was successful for the first tumour.

Different techniques in the field of molecular genetics have been used to elucidate tumour clonality, e.g. allelic imbalances [11, 12], CGH (comparative genomic hybridization) [13, 14], array comparative genomic hybridization (aCGH) [15, 16], as well as whole exome and whole genome sequencing [17–19]. In addition, several analytical tools have been proposed to justify the routine clinical use of determining tumour clonality [5, 13, 15, 20–22].

In the present study, 74 invasive breast tumours corresponding to 37 patients were stratified by laterality (bilateral vs. ipsilateral) and the time interval between the diagnosis of the first and second tumour (synchronous vs. metachronous). Both tumours from the same patient were analysed using several genome-wide screening methods and statistical approaches to assess tumour clonality. The level of concordance among the different statistical techniques and molecular data might help to define clonality in multiple tumours and guide treatment decisions for clinicians.

## Methods

### Patients and clinicopathological data

Fresh-frozen tumour specimens for 74 invasive breast carcinomas, corresponding to 37 patients diagnosed in Western Sweden between 1988 and 1998 with multiple breast cancers, were selected from the tumour bank at the Sahlgrenska University Hospital Oncology Lab (Gothenburg, Sweden). The patients were stratified into four groups based on the anatomic location of the multiple breast cancers (ipsilateral or bilateral) and time interval between the diagnoses (synchronous or metachronous). Ipsilateral was defined as tumours occurring in the same breast while bilateral was defined as the occurrence of tumours in both breasts. Metachronicity was defined as a time interval greater than 6 months between the diagnoses of the first and second tumours, while synchronicity specified that the two tumours occurred concurrently. Clinicopathological information was obtained from Regional Cancer Centre West (Gothenburg, Sweden) and the Sympathy and Melior databases (Sahlgrenska University Hospital). A part of the dataset was stratified into the molecular breast cancer subtypes (normal-like, basal-like, luminal subtype A, luminal subtype B/human epidermal growth factor receptor 2 (HER2)+, luminal subtype B/HER2-, and HER2/oestrogen receptor (ER)-) as described elsewhere [23, 24]. Luminal subtype B was further stratified according to HER2 status as determined by aCGH; HER2+ was set to log_2_ ratio ≥ + 0.5 and HER2- was set to log_2_ ratio < + 0.5 [25]. Routine haematoxylin and eosin-stained slides from formalin-fixed paraffin-embedded (FFPE) blocks were revised by a board-certified breast pathologist. Classification of the subtypes based on immunohistochemistry was not possible due to the lack of information on the Ki-67 status. The patients had an average follow-up time of 7.2 years. None of the patients were diagnosed with distant metastasis at the time of diagnosis of either the first or second tumours. The selection criteria were to use samples from opposite quadrants for ipsilateral cases and no nipple involvement. Representative imprints from each tumour specimen were stained with May-Grünwald Giemsa (Chemicon, Temecula CA, USA) and evaluated for neoplastic cells. Tumour specimens with at least 70% neoplastic cell content were included in downstream analyses.

### Array comparative genomic hybridization (aCGH) analysis

aCGH and data pre-processing was performed as previously described [24] and summarised in the Additional file 1: Supplementary Methods. Segmented data for segment analysis were generated using the “GLAD” package [26] in R (v3.4.3) [27]. The “Clonality” package [28] was used to define the likelihood ratio with individual comparisons (LR2) and LR2 *p* value and required copy number data procession with the “DNAcopy” package [29].

### DNA methylation analysis

Sixteen samples were randomly selected to represent each clinical group with four samples corresponding to two patients per group. Purified genomic DNA was processed at the SNP&SEQ technology platform, Uppsala, Sweden, using Illumina Infinium MethylationEPIC BeadChips (MethylationEPIC_v-1-0; mapped to UCSC Feb 2009 hg19: GRCh37). Raw data (IDAT files) were processed in R using the “RnBeads” package [30]. The probes were normalised with the BMIQ method (beta mixture quantile dilation) [31]. Beta values were obtained with “RnBeads”. The intensity values were extracted using the “ChAMP” package to generate segmented copy number data for the segment analysis [32, 33]. The “conumee” package was used to extract unsegmented information of CNAs on the probe level [34]. The unsegmented CNAs were used for the similarity index (SI), the distance measure and the clustering analysis.

### Whole transcriptome RNA sequencing (RNA-seq)

Total RNA samples were processed at the Science for Life Laboratory (National Genomics Infrastructure, Stockholm, Sweden). Illumina TruSeq strand-specific RNA libraries (Ribosomal depletion using RiboZero human) containing 125 bp paired-end reads were obtained for each sample on a HiSeq2000 sequencer (Illumina, San Diego, CA, USA). The computations were performed on resources provided by SNIC through Uppsala Multidisciplinary Center for Advanced Computational Science (UPPMAX) [35], as described in the Additional file 1: Supplementary Methods.

### Genome-wide single nucleotide polymorphism (SNP) genotyping analysis

Genome-wide SNP genotyping analysis was processed with Illumina Infinium HumanOmni2.5–8 v1.3 Beadchips at the SCIBLU Genomics DNA Microarray Resource Center (SCIBLU), Sweden, as described in the Additional file 1: Supplementary Methods.

### Statistical analyses

A *p* value cut-off of 0.05 was applied in all statistical tests.

#### Definition of tumour clonality

Tumours derived from a common precursor tumour cell should share certain features, i.e. similar CNAs, genetic variants, shared segments, DNA methylation and gene expression patterns, in addition to non-matching features that were acquired over time. We applied different statistical methods on different types of molecular data to identify similarities between the tumours that classify a tumour pair as clonal and reject the null hypothesis (different features due to independent development of primary tumours).

#### Similarity index (SI)

The SI assesses whether two tumours identified in the same patient are clonally related or two independent entities by identifying genetic aberrations that are patient-specific and non-recurrent aberrations frequently identified in cancer [21]. In brief, DNA copy number data were normalised and discretized (heterozygous loss (<− 0.3); normal; low-level gain (> 0.3)) and unique (*N*_*U*_), shared (*N*_*S*_), and opposite (*N*_*O*_) changes were calculated for each tumour pair to obtain the SI:

$$ SI=\frac{N_S}{N_S+{N}_U+{N}_O} $$

The SI ranges between 0 (completely different) and 1 (identical genomic profiles). The permutation-based *P*_*SI*_ gives the percentage of similarities between two tumours that are not due to recurrent chromosomal aberrations or randomness.

The SI remained unchanged for the gene expression microarray data. The normalised log_2_ ratios were discretized using a 1.5 fold change cut-off (underexpressed (log_2_ ratio < − 0.58); neutral; overexpressed (log_2_ ratio > 0.58)).

Calculation of the SI was modified for the methylation data (SI_met_) because the SI for copy number data is based on measuring the amount of alterations from the biologically neutral state (two copies per allele). In DNA methylation, neither methylated nor unmethylated can be defined as the neutral state of a cytosine due to the dynamic of methylation. The SI_met_ uses beta values discretized according to thresholds defined by Du et al. [36], where beta values > 0.8 are defined as methylated, and beta values < 0.2 as unmethylated, while the range from 0.2 to 0.8 is hemi-methylated. The SI_met_ counts the number of all common states between the first and the second tumour per probe and divides it by the total number of probes, giving the percentage of shared methylation states. The main difference is that the SI_met_ uses all probe states while the SI is based on the changes from the neutral state and therefore does not count two tumours that are normal as a shared state.

#### Hierarchical clustering

Unsupervised hierarchical clustering was applied using single linkage with Euclidean distance [37]. Clustering was performed using the basic “stats” package [27] for the aCGH-derived copy number data (imputed log_2_ ratios), the DNA methylation data (beta values and intensity values), the microarray-derived gene expression data (normalised log_2_ ratios), and the SNP array data (B allele frequency (BAF) and log R ratio (LRR) values). Two tumours of the same patient were defined as similar (clonal) if they clustered together in the terminal branch of the dendrogram.

#### Distance measure

The distance measure was used to compute the distance matrix of the Euclidean distances between different tumour samples to evaluate the similarity between two samples. The Euclidean distance was computed using the basic “stats” package [27] for the aCGH-derived copy number data (imputed log_2_ ratios), the DNA methylation data (beta values and intensity values), the microarray-derived gene expression data (normalised log_2_ ratios), and the SNP array data (LRR values). The distance measure was calculated for true tumour pairs which derive from the same patient and for all artificial combinations of tumour pairs from different patients (permutation). Tumour pairs that are more similar on the probe level will show a shorter distance from each other. Statistical significance for clonality was defined as the distance of a tumour pair of the same patient that is in the lower fifth percentile of the distribution of distances.

#### Shared segment analysis

In segmented copy number data, the breakpoints and the copy number of each segment was compared between the tumours. A shared segment was defined as an overlap of the exact loci in both ends of the segment where the change in status (loss or gain) occurred with the same direction (increase or decrease in copy numbers). The segment analysis was performed on segmented copy number data derived from aCGH (imputed log_2_ ratios), DNA methylation array (intensity values), and SNP array (LRR values). Shared segments were counted for true tumour pairs and all artificial pairs of the respective cohort. Clonality was defined as the number of shared segments above the 95^th^ percentile.

#### Mutational changes (genetic variants) and fusion transcript analysis

Mutational changes that were identical in both tumours were counted for true tumour pairs and all artificial pairs of the cohort. Clonality was defined as the number of shared mutations above the 95^th^ percentile of the permutation distribution. Shared mutations were counted for genomic and exonic RNA-seq data. In addition, a panel of 254 breast cancer and DNA repair-specific mutation spots proposed by Begg et al. was analysed [38]. The overlap of RNA-seq counts of the genomic and exonic data with the 254-gene panel was used to count the shared mutations of the true and artificial pairs of the cohort. Clonality was defined as the number of shared mutations above the 95^th^ percentile. To test for clonality using profiles of somatic mutations in the “Clonality” package [28], loci-specific probabilities of observing a mutation were obtained from the TCGA breast cancer dataset [39]. Furthermore, fusion transcripts of all tumours were compared and transcripts with identical 5′ and 3′ fusion partner breakpoints were counted.

#### Cohen’s kappa

Cohen’s kappa measures the chance-corrected agreement for two observations [40]. Cohen’s kappa indices of agreement between different methods applied to estimate clonality were calculated using the R-package “rel” [41].

## Results

### Tumour synchronicity strongly associated with metastatic spread to the axillary lymph nodes

The 37 breast cancer patients were stratified into four clinical groups based on tumour laterality and the time interval between the diagnoses of the first and second tumours (BM: bilateral-metachronous; BS: bilateral-synchronous; IM: ipsilateral-metachronous; IS: ipsilateral-synchronous). The clinicopathological characteristics are shown in Additional file 2: Table S1. Metastatic spread to the axillary lymph nodes was more prevalent in the synchronous groups (BS: 100%; IS: 85.7%) as compared to the metachronous groups (BM: 61.5%; IM: 14.3%; *P* = 0.001).

### Discordances in histopathological characteristics in 32% of the tumour pairs

For the clinical classification of clonality, several histopathological and molecular features were taken into consideration, including histological subtype, the status of ER and HER2, and the molecular subtype (Table 1). While the receptor status was available for most samples, the molecular subtype was only defined for about 40% of the tumours. Thirty-two percent of the patients (12/37) showed discordances between the first and the second tumour, with one-fourth of the 12 patients showing two discordant changes. Most changes were found in the histological subtypes (35%; 6/17 patients), while the molecular subtype differed in 25% (2/8 patients), ER status in 11% (4/35 patients), and HER2 status in 8% (3/37 patients). In patients with metachronous cancer, changes in receptor status from positive to negative were observed for patients BM6 and BM7. The discordant changes were equally distributed between the different clinical groups and showed no significance when stratified by group.

**Table 1:** Overview of the clinical and histological characteristics of the primary and secondary tumours

					Primary tumour				Secondary tumour					
Patient	Laterality	Synchronicity	Group	Time interval (days)	Histology	ER	HER2	Molecular subtype	Histology	ER	HER2	Molecular subtype	Discordance	Clinical classification
BM1	bilateral	metachronous	BM	346	Invasive carcinoma NST	pos	neg	ND	ND	pos	neg	ND		concordant
BM2	bilateral	metachronous	BM	1694	Invasive carcinoma NST	pos	neg	Luminal B	Invasive lobular carcinoma	pos	neg	ND	Histology	discordant
BM3	bilateral	metachronous	BM	1652	ND	pos	neg	ND	Invasive carcinoma NST	pos	neg	ND		concordant
BM4	bilateral	metachronous	BM	581	Invasive carcinoma NST	neg	neg	Basal-like	Invasive carcinoma NST	neg	neg	ND		concordant
BM5	bilateral	metachronous	BM	1954	Invasive carcinoma NST	pos	neg	ND	Invasive lobular carcinoma	pos	neg	ND	Histology	discordant
BM6	bilateral	metachronous	BM	1417	Invasive lobular carcinoma	pos	neg	ND	Invasive carcinoma NST	neg	neg	HER2/ER-	Histology; ER	discordant
BM7	bilateral	metachronous	BM	456	Invasive carcinoma NST	pos	pos	ND	Invasive carcinoma NST	neg	pos	ND	ER	discordant
BM8	bilateral	metachronous	BM	1152	Invasive lobular carcinoma	pos	neg	ND	Invasive lobular carcinoma	pos	neg	ND		concordant
BM9	bilateral	metachronous	BM	972	Invasive carcinoma NST	neg	neg	ND	Invasive carcinoma NST	neg	neg	ND		concordant
BS1	bilateral	synchronous	BS	0	Invasive carcinoma NST	pos	neg	ND	Invasive carcinoma NST	pos	neg	Luminal B		concordant
BS2	bilateral	synchronous	BS	0	Invasive lobular carcinoma	pos	neg	ND	Invasive carcinoma NST	pos	neg	Luminal B	Histology	discordant
BS3	bilateral	synchronous	BS	14	Invasive carcinoma NST	pos	neg	ND	Invasive carcinoma NST	pos	neg	ND		concordant
BS4	bilateral	synchronous	BS	0	ND	pos	neg	ND	ND	pos	neg	ND		concordant
BS5	bilateral	synchronous	BS	6	Invasive carcinoma NST	pos	neg	ND	Invasive carcinoma NST	ND	neg	ND		concordant
BS6	bilateral	synchronous	BS	0	ND	neg	neg	ND	ND	neg	neg	ND		concordant
BS7	bilateral	synchronous	BS	0	Invasive carcinoma NOS	pos	neg	Luminal B	Invasive lobular carcinoma	neg	neg	ND	Histology; ER	discordant
BS8	bilateral	synchronous	BS	0	Invasive carcinoma NST	pos	neg	ND	Invasive carcinoma NST	pos	neg	ND		concordant
IM1	ipsilateral	metachronous	IM	1855	ND	pos	neg	ND	ND	pos	neg	Luminal B		concordant
IM2	ipsilateral	metachronous	IM	448	ND	neg	neg	ND	ND	neg	neg	ND		concordant
IM3	ipsilateral	metachronous	IM	1944	Invasive carcinoma NST	pos	pos	Luminal B	ND	pos	pos	Luminal B		concordant
IM4	ipsilateral	metachronous	IM	567	Invasive carcinoma NST	pos	neg	Luminal B	ND	pos	neg	HER2/ER-	Subtype	discordant
IM5	ipsilateral	metachronous	IM	712	Invasive carcinoma NST	neg	neg	Basal-like	ND	neg	neg	ND		concordant
IM6	ipsilateral	metachronous	IM	664	ND	pos	neg	ND	ND	pos	neg	ND		concordant
IM7	ipsilateral	metachronous	IM	2454	ND	pos	neg	ND	ND	pos	neg	ND		concordant
IM8	ipsilateral	metachronous	IM	563	Invasive carcinoma NST	pos	neg	ND	Invasive lobular carcinoma	pos	neg	Luminal B	Histology	discordant
IM9	ipsilateral	metachronous	IM	2142	Invasive carcinoma NOS	ND	neg	Luminal B	Invasive carcinoma NOS	pos	neg	Luminal B		concordant
IS1	ipsilateral	synchronous	IS	0	Invasive carcinoma NST	neg	neg	Basal-like	Invasive carcinoma NST	neg	pos	Luminal B	HER2; subtype	discordant
IS2	ipsilateral	synchronous	IS	0	Invasive carcinoma NST	pos	neg	ND	ND	pos	neg	Luminal B		concordant
IS3	ipsilateral	synchronous	IS	0	ND	neg	neg	Basal-like	ND	neg	pos	Basal-like	HER2	discordant
IS4	ipsilateral	synchronous	IS	50	ND	neg	neg	ND	ND	pos	neg	Luminal B	ER	discordant
IS5	ipsilateral	synchronous	IS	0	Invasive carcinoma NST	pos	neg	Luminal B	Invasive carcinoma NST	pos	neg	Luminal B		concordant
IS6	ipsilateral	synchronous	IS	0	Invasive carcinoma NST	pos	neg	ND	ND	pos	neg	ND		concordant
IS7	ipsilateral	synchronous	IS	0	Invasive carcinoma NOS	pos	neg	ND	ND	pos	neg	ND		concordant
IS8	ipsilateral	synchronous	IS	0	Invasive carcinoma NST	pos	neg	Luminal B	ND	pos	neg	ND		concordant
IS9	ipsilateral	synchronous	IS	0	ND	neg	pos	Basal-like	ND	neg	neg	ND	HER2	discordant
IS10	ipsilateral	synchronous	IS	0	Invasive carcinoma NST	pos	neg	Luminal B	ND	pos	neg	Luminal B		concordant
IS11	ipsilateral	synchronous	IS	0	Invasive carcinoma NOS	neg	pos	HER2/ER-	ND	neg	pos	HER2/ER-		concordant

*ER* oestrogen receptor status, *HER2* human epidermal growth factor receptor 2 status, *ND* not determined, *NOS* not otherwise specified, *NST* no special type

### Stratification by laterality revealed differential copy number imbalances

**Fig. 1:**
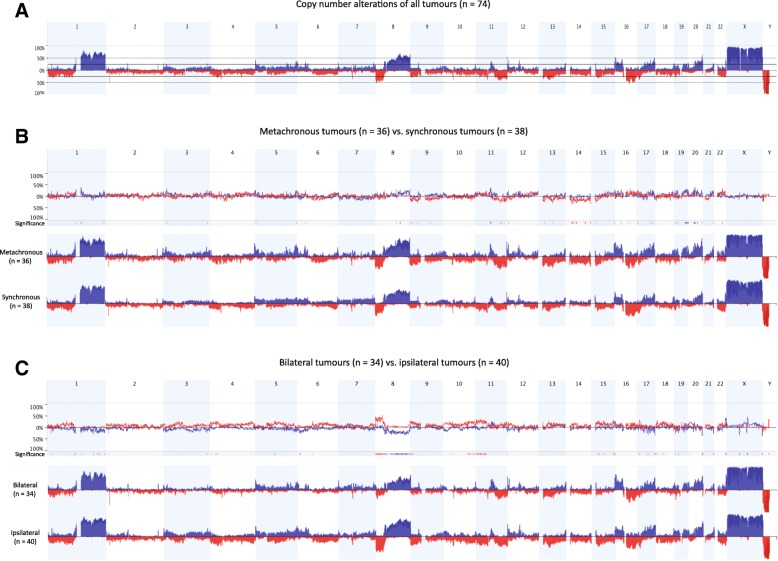
Genome-wide frequency plots of DNA copy number gains (*blue*) and losses (*red*) for the entire cohort (**a**), as well as cohorts stratified by the time interval between the tumours (**b**; metachronous (*n* = 36) vs. synchronous (*n* = 38)) and the laterality (**c**; bilateral (*n* = 34) vs. ipsilateral (*n* = 40))

DNA copy number analysis using aCGH was performed to identify recurrent regions of DNA copy number gain (blue) and loss (red) in at least 25% of the tumours in the patient cohort. Recurrent DNA gains were identified on chromosomes 1q, 8q, 16p, 17q, and 20q, while DNA loss was detected on 1p, 8p, 11q, 13q, and 16q (Fig. 1a). These results were in line with DNA gains and losses frequently identified in breast cancer [42–44]. There was very little difference in the DNA copy number profiles when stratified by synchronicity (excluding copy number variations (CNVs) and probes from sex chromosomes) with 59 significantly different genomic regions displaying DNA copy number imbalances (Fig. 1b). Most noticeable were losses of the entire chromosome 14 and the long arm of chromosome 11 in the metachronous subgroup. In contrast, stratification by laterality yielded 134 statistically significant minimal common regions of copy number imbalances, including more fractions of genome altered in the ipsilateral subgroup with prominent losses on 8p and 11p (Fig. 1c).

### DNA methylation showed higher variability in synchronous tumours

**Fig. 2:**
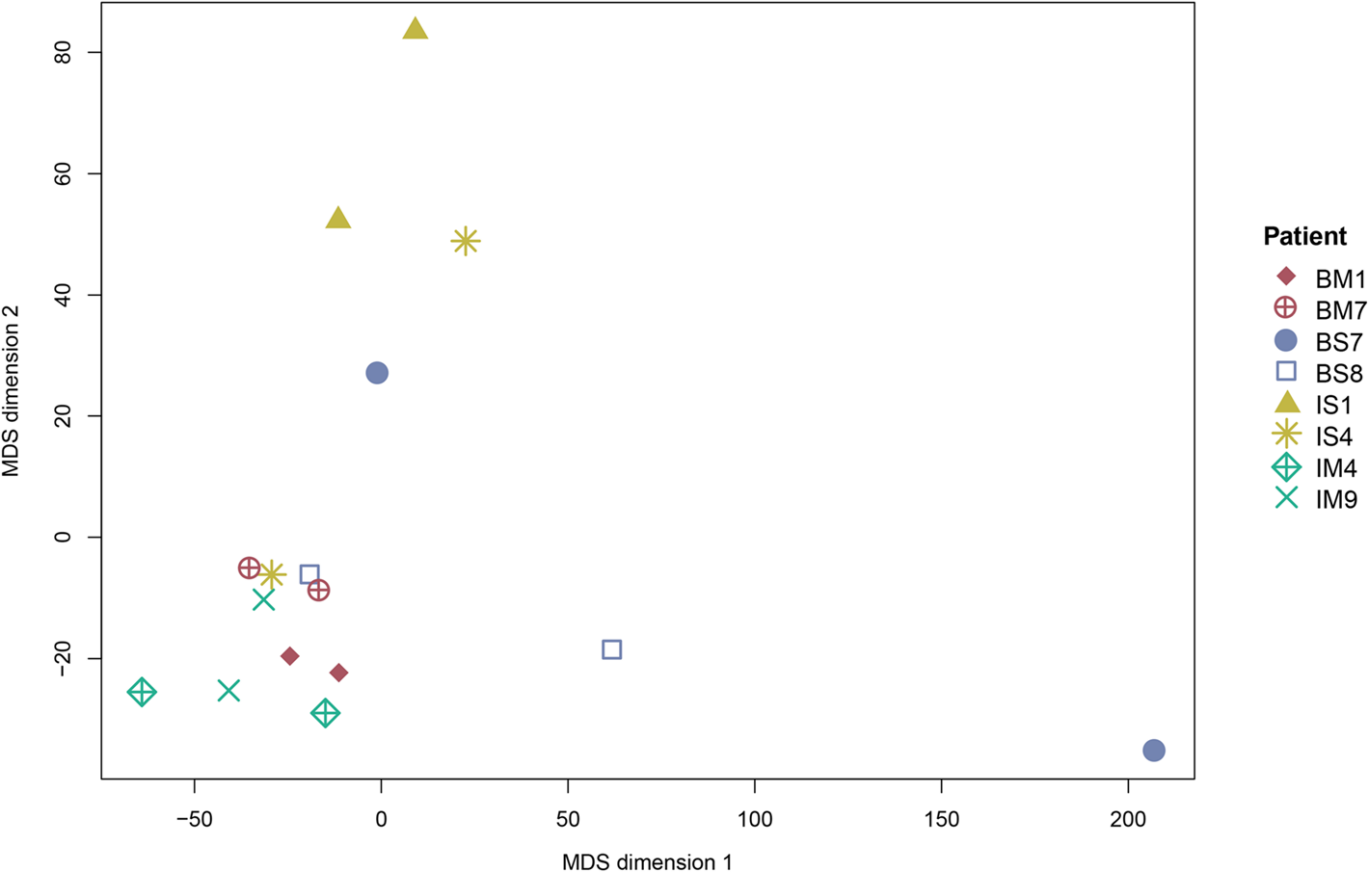
Kruskal’s non-metric multidimensional scaling (MDS) plot of beta values from the DNA methylation cohort (*n* = 16). The MDS plot visualised similarities between the individual samples based on information from the distance matrix

The variability of the beta values was the highest in the bilateral and synchronous groups and consequently in the BS group, which was in line with patients BS7 and BS8 having the highest variability in methylation patterns between the two respective tumour pairs (Additional file 3: Table S2). Principal component analysis of the methylation data showed a statistically significant association with synchronicity (*P* = 0.007), while no further associations to other variables were found. Kruskal’s non-metric multidimensional scaling (MDS) demonstrated that most of the synchronous samples were further away from each other, while the metachronous samples formed a distinct cluster, suggesting a higher variability of beta values in synchronous samples (Fig. 2).

Strong consensus in clonality could be found for the tumours of patients BM7, BS8, and IS1, while the tumour pairs for patients BS7 and IS4 were determined to be independent primary tumours (Table 2). In general, DNA methylation intensity values were a more liberal method for clonality classification, in particular the clustering analysis, and frequently classified tumour pairs as similar in comparison with other types of molecular data.

**Table 2:** Summary of clonality tests for the methylation cohort (*n* = 8)

	Patients	BM1	BM7	BS7	BS8	IM4	IM9	IS1	IS4
	Laterality	Bilateral	Bilateral	Bilateral	Bilateral	Ipsilateral	Ipsilateral	Ipsilateral	Ipsilateral
	Synchronicity	Metachronous	Metachronous	Synchronous	Synchronous	Metachronous	Metachronous	Synchronous	Synchronous
	Group	BM	BM	BS	BS	IM	IM	IS	IS
aCGH data	**Similarity Index**								
	SI	0.261	0.580	0.323	0.571	0.402	0.237	0.217	0.200
	*P*_SI_	25.600	66.450	39.810	65.930	51.650	17.890	10.510	2.710
	*P*	0.183	**0.005**	0.087	**0.005**	**0.032**	0.269	0.359	0.434
	**Clustering (Euclidean distance, single linkage)**								
	Clustering	different	**similar**	different	**similar**	**similar**	different	different	different
	**Distance measure**								
	Euclidean	46.801	19.823	59.036	16.362	43.821	60.220	63.932	66.099
	5th percentile	not sign.	**significant**	not sign.	**significant**	not sign.	not sign.	not sign.	not sign.
	**Shared segments**								
	Segments	34	37	10	32	24	3	53	38
	95th percentile	**significant**	**significant**	not sign.	**significant**	**significant**	not sign.	**significant**	**significant**
	**Clonality package**								
	LR2	0.044	135.409	0.011	34,945,440	0.008	0.006	0.000	0.001
	*P*	0.262	**0.009**	0.455	**0.000**	0.519	0.552	0.912	0.879
Methylation data Beta values	**Similarity Index for methylation**								
	SI_met_	0.879	0.880	0.685	0.815	0.868	0.871	0.911	0.833
	*P*_SI_	8.510	8.610	0.000	1.240	7.350	7.660	11.710	3.410
	*P*	**0.018**	**0.018**	0.947	0.526	**0.018**	**0.018**	**0.018**	0.333
	**Clustering (Euclidean distance, single linkage)**								
	Clustering	**similar**	different	different	different	**similar**	different	**similar**	different
	**Distance measure**								
	Euclidean	87.969	86.972	228.809	125.860	92.508	90.574	71.300	134.247
	5th percentile	**significant**	**significant**	not sign.	not sign.	not sign.	not sign.	**significant**	not sign.
Methylation data Intensity values	**Similarity Index**								
	SI	0.578	0.573	0.439	0.594	0.517	0.565	0.648	0.467
	*P*_SI_	20.360	19.770	0.000	22.520	11.010	18.650	29.070	1.410
	*P*	**0.018**	**0.018**	0.737	**0.018**	0.088	**0.018**	**0.018**	0.456
	**Clustering (Euclidean distance, single linkage)**								
	Clustering	**similar**	**similar**	different	**similar**	**similar**	**similar**	**similar**	**similar**
	**Distance measure**								
	Euclidean	148.829	130.060	194.202	158.604	160.988	147.229	124.268	171.320
	5th percentile	**significant**	**significant**	not sign.	**significant**	**significant**	**significant**	**significant**	not sign.
	**Shared segments**								
	Segments	64	14	9	18	6	11	47	7
	95th percentile	**significant**	**significant**	not sign.	**significant**	not sign.	not sign.	**significant**	not sign.
	**Clonality package**								
	LR2	4.53	28.46	0.00	241,461,300	300,802.20	1.63	34,907,040,000,000	0.15
	*P*	0.080	**0.036**	0.795	**0.000**	**0.009**	0.107	**0.000**	0.241
**Consensus in clonality:**		7/13	12/13	0/13	10/13	8/13	4/13	9/13	2/13

*P*_*SI*_ percentage of similarities between two tumours that are not due to recurrent chromosomal aberrations or randomness, *LR2* final likelihood ratio with individual comparisons. Statistically significant variables (*P* < 0.05) are displayed in bold text

### Ipsilateral synchronous tumours showed similar gene expression by microarray

The gene expression cohort consisted of seven patients with ipsilateral tumours (three metachronous and four synchronous). The clonality analyses based on gene expression microarray data showed strong concordance to the clinical groups with all four synchronous cases being similar for all analyses while 2/3 metachronous cases were classified as different entities (Additional file 4: Table S3). All analyses of the gene expression cohort were in line with the aCGH results except for patient IM4, whom was classified as independent in the gene expression analysis and equivocal in the aCGH data set. MDS demonstrated similar gene expression patterns between the tumour pairs of the patients IM3, IS3, and IS10 (Additional file 5: Figure S1A).

### Varying tendencies for clonality within RNA-seq and SNP data

**Fig. 3:**
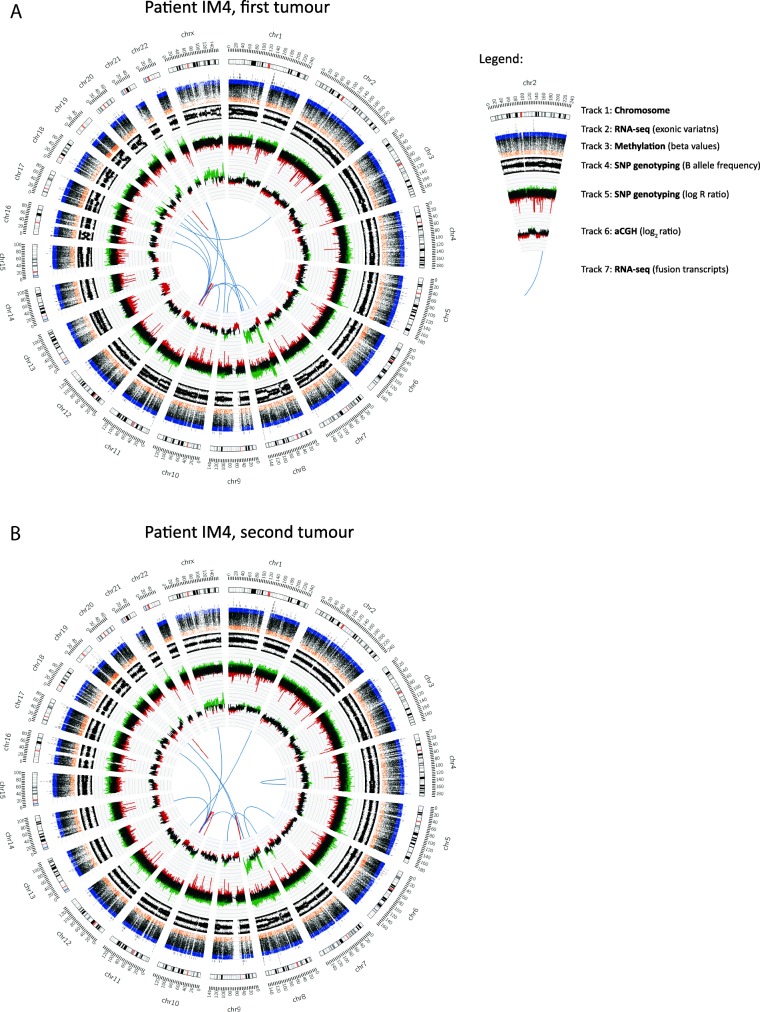
Circos plots depicting aCGH-derived DNA copy number profiles, genome-wide SNP genotyping, DNA methylation beta values, and RNA-seq data in the first (**a**) and second (**b**) tumour of breast carcinoma patient IM4. Circos plot *Track 1:* Chromosome cytobands from pter to qter. The centromere is shown as a *red bar*. *Track 2:* Mutations in exonic regions (exonic variants) identified with RNA-seq data are shown as *dark grey bars*. *Track 3:* Beta values of DNA methylation data. *Track 4:* B allele frequency of SNP genotyping data. *Track 5:* Log R ratio of SNP genotyping data, where copy number gains and losses are depicted in *green* and *red*, respectively. *Track 6:* Log_2_ ratio of aCGH data, where copy number gains and losses are depicted in *green* and red, respectively. *Track 7:* Gene fusions identified with RNA-seq data. Intrachromosomal and interchromosomal gene fusions are shown in *red* and *blue lines*, respectively

RNA-seq and SNP genotyping were performed for both tumours of patients IM4, IS10, and IS11. A total of 64 fusion transcripts were detected in the two tumours of patient IM4, with five fusion transcripts (7.8%) containing the same fusion breakpoints in the 5′- and 3′-gene partners in both tumours (Additional file 6: Table S4). For patients IS10 and IS11, 1/836 (0.1%) and 5/153 (3.3%) fusion transcripts were identical between the two tumours, respectively. No other shared fusion transcripts were found between different tumours. The RNA-seq data was then evaluated to identify shared genetic variants in genomic and exonic (coding) regions. Shared genomic variants (genome-wide and the 254-gene panel) showed similar tendencies that were in line with the aCGH distance measure and SNP shared segment (LRR) data (Additional file 7: Table S5). The shared exonic variants in the 254-gene panel only found two shared mutations in patient IM4, which contradicted most other RNA-seq results. The shared segment and clustering analyses of the SNP array data classified patient IS10 as clonal, which was in line with the aCGH results but contradicted the distance measure and MDS, which classified the LRRs of the tumour pair IM4 as most similar (Additional file 5: Figure S1B). The “Clonality” package applied on the exonic variants classified all tumour pairs as clonal. A circos plot summarising the results of patient IM4 visualised the similarities in copy number profiles of both aCGH-derived log_2_ ratio and SNP array-derived LRR and fusion transcripts (Fig. 3).

### Tumour clonality defined in 46% of the patients

**Fig. 4:**
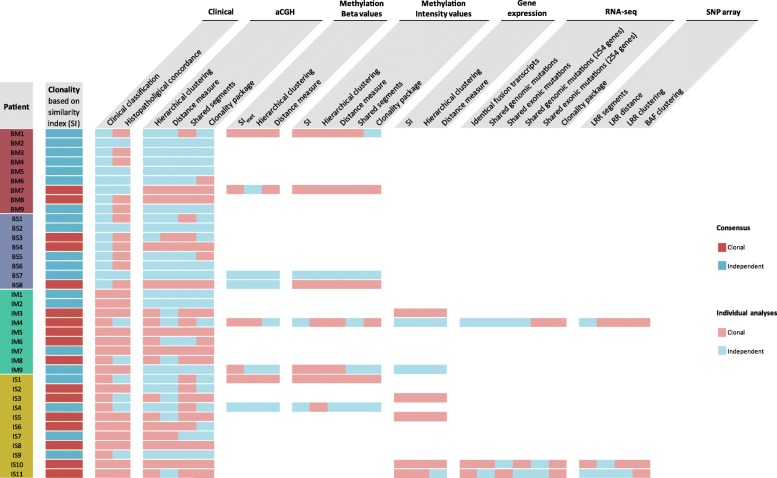
Overview of the different statistical methods applied sorted by the type of data. *Red boxes* indicate that the analysis defined the tumour pair as clonal and *blue boxes* indicate independence of the tumours. *BAF* B allele frequency, *BM* bilateral-metachronous, *BS* bilateral-synchronous, *IM* ipsilateral-metachronous, *IS* ipsilateral-synchronous, *LRR* log R ratio, *SI* similarity index, *SI*_*met*_ modified SI for methylation data

Calculation of Cohen’s kappa indices was applied to detect the highest agreement between the different statistical methods used to estimate clonality. For the aCGH data, hierarchical clustering and the similarity index (SI) were identified as the most appropriate (0.659 and 0.630, respectively). Since the SI is easier to interpret as a measure and independent of the cohort, it presented the most reasonable definition of clonality and determined 46% (17/37) of the tumour pairs as clonal (Fig. 4). No statistical significance was found to associate the tumour clonality defined by the SI with the clinical classification (Wilcoxon rank sum test: *P*_Laterality_ = 0.247; *P*_Synchronicity_ = 0.095; Analysis of variance (ANOVA): *P*_Clinical groups_ = 0.229), highlighting the alarming reality that there is very little connection between current clinical guidelines and the biology underlying tumour clonality.

The majority of the analyses conducted were in agreement with the SI except for patients BM1, IM4, IM7, IS1, and IS7 (Fig. 4). Interestingly, the histopathological concordances often showed opposite tendencies compared to the aCGH analysis. The different methods applied to the DNA methylation, gene expression and SNP array data sets displayed strong homogeneity within their type of data regardless of the method applied. The results for the SI and hierarchical clustering were consistent in most data sets. The distance measure also overlapped with these results but seemed to be a more conservative measure since fewer tumour pairs were classified as clonal. The shared segment analysis with the aCGH data clearly favoured the clonality hypothesis with defining 21/37 tumour pairs as clonal along with 4/8 cases in the methylation intensity data and 1/3 in the LRR data. The shared segment analysis was in most cases consistent between the different types of data.

## Discussion

Here, we show that molecular and statistical analyses are powerful tools for classifying clonal recurrences and independent primary tumours. This study provides valuable insight into which molecular technologies were most informative for investigating clonal relatedness in tumour pairs. Although tumour clonality should govern the choice of treatment, bilateral breast tumours are generally treated as different primary tumours and not as potential failure of the previous treatment. Tumour characteristics such as histological subtype, molecular subtype, presence of ductal carcinoma in situ (DCIS), and receptor status are currently used to choose treatment strategies for patients with multiple breast tumours. However, to fully comprehend the association between multiple tumours, routine clinical and diagnostic testing needs to be conducted in conjunction with molecular and bioinformatics methods.

In the majority of the analyses, the type of molecular data analysed had a stronger impact on clonality determination than the analytical method used. This raises the question of which biological phenomenon provides the most stable evidence for clonality. DNA methylation and gene expression are more dynamic than DNA mutations and CNAs, and might therefore be more similar due to environmental factors. CNAs are acquired at early stages of tumourigenesis [45, 46] making them the most stable type of biological data in this study. An overlap of tendencies in clonality between the aCGH and DNA methylation data was seen for only 50% of the cohort (BM7, BS7, BS8, and IS4), giving a less optimistic view on using DNA methylation as a clonality tool compared to results from other reported studies [47, 48]. In the DNA methylation data, synchronicity accounted for more variation than metachronicity, providing further evidence that synchronous tumours are more different from each other with regard to DNA methylation patterns. However, the small cohort size limited the conclusions that can be drawn. The overlap of results between gene expression and copy number data was surprisingly high since gene expression is more unstable than DNA alterations. Gene expression-based analyses defined all IS cases as clonal indicating that gene expression patterns are very similar for tumour cells arising in the same breast at the same time, possibly due to their adjacent microenvironment.

Hierarchical clustering has been used, among other methods, in several studies to define clonality [15, 47, 49]. Clustering is designed as an unsupervised classification tool to discover underlying structures of a data set under the assumption that the number of clusters and their members are unknown. The disadvantage of clustering is that clonality depends on the relationship between individual tumours and the linkage between tumour clusters. Using Euclidean distance with single linkage is the only way to circumvent these disadvantages [37]. The results from the SI and hierarchical clustering analyses exhibited a strong overlap in their classification. Calculation of Cohen’s kappa showed the highest agreement of the different analyses for the SI and the clustering. Thus, the SI represented the most suitable approach in defining clonality since it is a specialised technique specifically developed for this purpose and provides easy interpretation.

In the DNA methylation cohort, clustering of the intensity values classified 7/8 tumour pairs as clonal and therefore did not provide a precise segregation between clonally related tumours and independent tumours. The aCGH, DNA methylation intensity and LRR data should biologically refer to the same phenomenon (CNAs) and consequently show the same tendencies for different genomic loci. Therefore, it was unexpected that the results of the clustering and shared segment analysis for those data sets did not show stronger concordance. Furthermore, it was anticipated that the results from the clustering and the distance measure were more in agreement since the first step of clustering is the Euclidean distance. In most cases, the distance measure seemed to be a stricter method than the SI and clustering.

In comparison with genomic variants, mutation analyses based on exonic variants or gene panels represent a subset of the full picture. The different tendencies between the methods represent a drawback for potential applications of sequencing panels in the clinic. The fusion transcript analysis was the only method that did not show any overlaps between patients. Moreover, unspliced fusion transcripts provide the transcribed level of CNAs, which highlights the functional consequences of CNAs and makes them an important tool to assess tumour clonality. Our RNA-seq-based mutation approach had several limitations starting with the lack of matched normal samples to exclude germline mutations and normal DNA nucleotide variations. However, common genetic variants found in the human population were removed. Furthermore, our approach did not account for the frequency of mutations in breast cancer since rare mutations give much stronger evidence for clonality than common mutations [22]. In the frequency-based approach of the “Clonality” package, a further limitation was that RNA-seq data was compared with whole exome sequencing data from TCGA. In addition, the RNA-seq cohort was too small to perform meaningful statistics regarding the 95th percentile, which is a general limitation of using permutation-based approaches. Therefore, the results from this cohort have to be viewed with caution and in context to the other results. Tumours from patient IS10, for example were clonal regarding all other analyses except the RNA-seq and SNP genotyping array.

Whole genome sequencing (WGS) is the more appropriate method to evaluate mutations in comparison with RNA-seq, which does not give information on untranscribed DNA sequences. Hence, the lack of common mutations cannot be considered as a guarantee that tumour pairs are independent. However, intratumour heterogeneity complicates clonality analyses due to biological differences in different parts of a tumour and subclone evolution. In aCGH, contamination with normal cells could diminish the intensity of detected CNAs and small cell populations might not be detected. However, by using only samples that showed a tumour cell content of at least 70%, we ruled out that a lack of clonal relatedness could be due to a lack of tumour cells.

Few studies based on molecular approaches have been conducted to define clonality in multiple breast tumours and there is no consensus on which type of data and analysis method provides the most stable definition of clonality. A direct comparison of these studies to the findings presented here might, however, not be justified due to differences in the study set-up, methods and statistics. In a study on a contralateral cohort using low-coverage WGS, Alkner et al. demonstrated clonal relatedness in 10% (1/10) of the patients [19], which was lower than the clonal relatedness of bilateral tumours in our study (29%, 5/17 patients). Klevebring et al. found 12% (3/25) of their BM cohort to be clonally related using whole exome sequencing (WES) [18], which was also lower than the clonal relatedness of BM tumours in our study (22%, 2/9 patients). Desmedt et al. studied IS tumours and defined 67% (24/36) of the patients as clonal using a targeted mutation screening and 100% (8/8) of the patients as clonal using low-coverage WGS [50]. Our IS cohort showed clonality in 64% (7/11) of the patients, which is surprisingly closer to the mutational approach than the copy number-based approach. Our report is the first, to our knowledge, to compare different approaches (type of molecular data and statistical method) and clinical groups (BM, BS, IM, and IS) between each other.

## Conclusions

There are many studies published on tumour clonality using different types of data and statistical methods. Most studies defined their own methods and cohort-specific cut-offs. Currently, there is no consensus about which type of data and especially which statistical analysis is the most suitable and there are surprisingly few studies that compare and evaluate the feasibility of these different approaches. Nonetheless, extremely similar or different tumour pairs (BM7, BS7, IM3, IS4, and IS5) showed consistent results regardless of the statistical analysis or biological data used, but clinic guidelines need to be defined with exact thresholds in order to standardise clonality testing in a routine diagnostic setting. In metachronous cancer, clonality between the first and second tumour may indicate an insufficient effect of the treatment for the first tumour and the patient could benefit from a change in treatment. An independent new primary tumour would indicate a more favourable prognosis than a recurrence. Hence, the discrimination between a clonal and independent origin of the second tumour is of high importance for the patient. In our study, the distance measure proved to be the most conservative method for defining clonality and the shared segment analysis the most liberal. Gene expression data classified all ipsilateral-synchronous cases as clonal, demonstrating that gene expression strongly depends on the nearby tumour microenvironment. The SI using aCGH data was found to be the most suitable method to classify tumour clonality, as it had the highest concordance with all results and can be easily integrated into clinic routine using FFPE samples to obtain copy number data. But most importantly, the definition of tumour clonality based on the current clinicopathological markers needs to be revised due to the limited intersects between current clinical guidelines and the underlying biology of tumour clonality.

## Additional files

Additional file 1: Supplementary Methods**.** Description of nucleic acid isolation and purification, *aCGH* gene expression microarray, *RNA-seq* and SNP array analysis. (DOCX 37 kb)

Additional file 2: **Table S1.** Overview of clinical characteristics of the patient and tumour information stratified by the clinical groups (BM, BS, IM, and IS). (XLSX 16 kb)

Additional file 3: **Table S2.** Variabilities of the studied sample groups with the variability spanning between 5th and 95th percentile of the beta values. (XLSX 10 kb)

Additional file 4: **Table S3.** Summary of clonality tests for the gene expression microarray cohort (*n* = 7). (XLSX 14 kb)

Additional file 5: **Figure S1.** Non-metric multidimensional scaling (MDS) plot of (**A**) normalised log_2_ ratios from gene expression data, and (**B**) LRR values from SNP array data. The MDS plot visualised similarities between the individual samples based on information from the distance matrix. (TIF 1784 kb)

Additional file 6: **Table S4.** Overview of the shared fusion transcripts in patient IM4, IS10, and IS11. (XLSX 13 kb)

Additional file 7: **Table S5.**Summary of clonality tests for the RNA-seq and SNP genotyping cohort (*n* = 6). (XLSX 14 kb)

## Abbreviations

aCGH: Array comparative genomic hybridization
ANOVA: Analysis of variance
BAF: B allele frequency
BM: bilateral-metachronous
BS: bilateral-synchronous
CGH: Comparative genomic hybridization
CNA: Copy number alteration
CNV: Copy number variation
DCIS: Ductal carcinoma in situ
ER: Oestrogen receptor status
FFPE: Formalin-fixed paraffin-embedded
HER2: Human epidermal growth factor receptor 2 status
IM: Ipsilateral-metachronous
IS: Ipsilateral-synchronous
LR2: Likelihood ratio with individual comparisons
LRR: Log R ratio
MDS: Kruskal’s non-metric multidimensional scaling
NOS: Not otherwise specified
NST: No special type
RNA-seq: RNA sequencing
SI: Similarity index
SI_met_: Modified SI for methylation data
SNP: Single nucleotide polymorphism
WES: Whole exome sequencing
WGS: Whole genome sequencing
