# Supplementary material for: Clonal relatedness in tumour pairs of breast cancer patients
Source: Breast Cancer Res. 2018 Aug 9;20:96. doi: 10.1186/s13058-018-1022-y (PMC6085699; doi:10.1186/s13058-018-1022-y)
Supplement: Supplementary file 1 — Supplementary Methods. Description of nucleic acid isolation and purification, aCGH gene expression microarray, RNA-seq and SNP array analysis. (DOCX 37 kb) [file 13058_2018_1022_MOESM1_ESM.docx]

**Supplementary Methods**

**Nucleic acid isolation and purification**

For Array Comparative Genomic Hybridization (aCGH), SNP genotyping, DNA methylation array analysis, gene expression microarray analysis, and RNA sequencing (RNA-seq) analysis, genomic DNA and total RNA were isolated from 10‐20 mg sections of fresh‐frozen tumour specimens. Prior to nucleic acid isolation, each specimen was evaluated for neoplastic cell content using touch preparation imprints stained with May-Grünwald Giemsa (Chemicon) including only specimens with at least 70% neoplastic cell content in downstream analyses. Genomic DNA was isolated using the Wizard Genomic DNA extraction kit (Promega), including proteinase K treatment (Roche) followed by phenol‐chloroform purification (Sigma). Total RNA was isolated with the RNeasy Lipid Tissue Mini Kit (Qiagen) according to the manufacturer’s instructions. DNA and RNA concentration were measured using Nanodrop ND‐1000 (Nanodrop Technologies). The total RNA concentration was also evaluated using QuBit (ThermoFisher Scientific) while RNA integrity was assessed using the RNA 6000 Nano LabChip Kit with Agilent 2100 Bioanalyzer (Agilent Technologies).

**Array Comparative Genomic Hybridization (aCGH)**

Whole‐genome tiling arrays with 38,043 BAC reporters (UCSC May 2004 hg17: NCBI Build 35) were manufactured as previously described [1] at the SCIBLU Genomics DNA Microarray Resource Center, Lund, Sweden. The clone set consisted of the 32K BAC clone library (BacPac Resources), the 3.4K FISH Mapped Clones Version 1.3 (BacPac Resources), clones located in telomeric regions [2], and clones covering microdeletion syndromes [3]. Male genomic DNA was used as a reference for the aCGH data. Images and raw signal intensities were acquired using an Agilent G2505B DNA microarray scanner (Agilent Technologies) and GenePix Pro 6.0.1.22 (Axon Instruments) image analysis software. Data preprocessing and pin‐based Lowess normalization were performed using the web‐based BioArray Software Environment system [4, 5] provided by SCIBLU. Segmentation into regions of gains and losses was performed using the Rank Segmentation algorithm with Nexus Copy Number Professional 7.5 software (BioDiscovery; settings: 5.0E‐5 significance threshold, 1000 kb maximum contiguous probe spacing, minimum of 5 probes per segment). Log_2_ ratio thresholds for low-level gain and heterozygous loss were set at +0.3 and -0.3, respectively. Minimal common regions of copy number imbalances were identified when observed in at least 25% of the tumour samples with a CNV overlap <99%.

**Gene expression microarray**

Illumina HumanHT-12 gene expression profiles for 14 tumours (seven tumour pairs) were selected for a previous study on DNA amplification of the 8p11-p12 genomic region and evaluated as previously described [6]. In brief, data pre-processing and quantile normalisation were applied to the raw signal intensities using the web-based BioArray Software Environment system [4] provided by Swegene Genomics DNA Microarray Resource Center (SCIBLU). Further data processing was performed in Nexus Expression 2.0 (BioDiscovery) using log_2_-transformed, normalised expression values and a variance filter.

**Whole transcriptome RNA sequencing (RNA-seq)**

Total RNA samples from six tumours (three tumour pairs) that were selected from a previous study on DNA amplification of the 8p11-p12 genomic region [6] were processed at the SciLifeLab (National Genomics Infrastructure Stockholm). Illumina TruSeq strand-specific RNA libraries (Ribosomal depletion using RiboZero human) containing 125 bp paired-end reads were obtained for each sample on a HiSeq2000 sequencer (Illumina).

*Quality control*

Quality control of raw RNA-seq reads was performed prior to assembly using FastQC (0.11.5). The RNA-seq reads were then trimmed and filtered with TrimGalore (0.3.3) to remove adapter sequences and reads with Phred quality scores below 20, followed by alignment to the hg19 build 37 reference assembly of the human genome using STAR (2.5.1b) [7]. Read alignment yielded approximately 40-50 million aligned reads per sample. Counts and Fragments Per Kilobase of transcript per Million mapped reads (FPKM) were calculated using HtSeq (0.6.1) [8] and Cufflinks (2.2.1) [9], respectively. Quality control statistics for mapped reads (*e.g.* gene body coverage and read distribution) were obtained using RSeQC (2.3.6).

*Fusion gene identification*

Fusion transcripts were identified with FusionCatcher (0.99.5a) using criteria to remove false positive candidate fusion events, followed by classification of “driver” fusion events (Bayesian probability scores <0.5) with oncogenic potential using Oncofuse (1.1.1) [10, 11].

*Variant calling and filtering*

The Genome Analysis Toolkit (GATK 3.5.0) variant calling pipeline [12] and the ANNOVAR tool (2016.05.11) were used to identify and annotate genetic variants, *e.g.* SNPs and indels, in individual samples with the SplitNCigarReads, BaseRecalibrator (with dbSNP Build 138 hg19), HaplotypeCaller, and VariantFiltration tools, respectively. Common genetic variants found in the human population were removed with ANNOVAR using the dbSNP (hg19_snp138) and 1000 Genomes Project databases (1000g2015aug) with a minor allele frequency (MAF) threshold of 0.01.

**Genome-wide SNP genotyping analysis**

Genome-wide SNP genotyping analysis for six tumours (three tumour pairs; selected for DNA amplification of the 8p11-p12 genomic region [13]) was processed with Illumina Infinium HumanOmni2.5-8 v1.3 Beadchips at the SCIBLU Genomics DNA Microarray Resource Center (SCIBLU), Lund University, Sweden. The beadchips were scanned on an iScan (Illumina) and data processed using the Illumina GenomeStudio Genotyping Module software (V2011.1) and hg19 build 37 reference assembly of the human genome to calculate B-allele frequencies (BAF) and logR ratios (LRR). Circos plots were generated with the Circos module (0.66) to visualize fusion genes, DNA copy number alterations, SNP-derived copy number profiles, methylation beta values, and exonic variants for each sample [14].

**References**

1. Jonsson G, Staaf J, Olsson E, Heidenblad M, Vallon-Christersson J, Osoegawa K, de Jong P, Oredsson S, Ringner M, Hoglund M *et al*: **High-resolution genomic profiles of breast cancer cell lines assessed by tiling BAC array comparative genomic hybridization**. *Genes Chromosomes Cancer* 2007, **46**(6):543-558.

2. Knight SJ, Lese CM, Precht KS, Kuc J, Ning Y, Lucas S, Regan R, Brenan M, Nicod A, Lawrie NM *et al*: **An optimized set of human telomere clones for studying telomere integrity and architecture**. *Am J Hum Genet* 2000, **67**(2):320-332.

3. Vissers LE, de Vries BB, Osoegawa K, Janssen IM, Feuth T, Choy CO, Straatman H, van der Vliet W, Huys EH, van Rijk A *et al*: **Array-based comparative genomic hybridization for the genomewide detection of submicroscopic chromosomal abnormalities**. *Am J Hum Genet* 2003, **73**(6):1261-1270.

4. **BASE - BioArray Software Environment** [<http://base.thep.lu.se/>]

5. Saal LH, Troein C, Vallon-Christersson J, Gruvberger S, Borg A, Peterson C: **BioArray Software Environment (BASE): a platform for comprehensive management and analysis of microarray data**. *Genome Biol* 2002, **3**(8):SOFTWARE0003.

6. Parris TZ, Danielsson A, Nemes S, Kovacs A, Delle U, Fallenius G, Mollerstrom E, Karlsson P, Helou K: **Clinical implications of gene dosage and gene expression patterns in diploid breast carcinoma**. *Clin Cancer Res* 2010, **16**(15):3860-3874.

7. Williams CR, Baccarella A, Parrish JZ, Kim CC: **Trimming of sequence reads alters RNA-Seq gene expression estimates**. *BMC Bioinformatics* 2016, **17**:103.

8. Anders S, Pyl PT, Huber W: **HTSeq--a Python framework to work with high-throughput sequencing data**. *Bioinformatics* 2015, **31**(2):166-169.

9. Trapnell C, Roberts A, Goff L, Pertea G, Kim D, Kelley DR, Pimentel H, Salzberg SL, Rinn JL, Pachter L: **Differential gene and transcript expression analysis of RNA-seq experiments with TopHat and Cufflinks**. *Nat Protoc* 2012, **7**(3):562-578.

10. Nicorici D. , Satalan M. , Edgren H. , Kangaspeska S. , Murumagi A. , Kallioniemi O. , Virtanen S. , O. K: **FusionCatcher – a tool for finding somatic fusion genes in paired-end RNA-sequencing data**. *bioRxiv* 2014.

11. Shugay M, Ortiz de Mendibil I, Vizmanos JL, Novo FJ: **Oncofuse: a computational framework for the prediction of the oncogenic potential of gene fusions**. *Bioinformatics* 2013, **29**(20):2539-2546.

12. McKenna A, Hanna M, Banks E, Sivachenko A, Cibulskis K, Kernytsky A, Garimella K, Altshuler D, Gabriel S, Daly M *et al*: **The Genome Analysis Toolkit: a MapReduce framework for analyzing next-generation DNA sequencing data**. *Genome Res* 2010, **20**(9):1297-1303.

13. Parris TZ, Biermann J, Engqvist H, Werner Rönnerman E, Truvé K, Nemes S, Forssell-Aronsson E, Solinas G, Kovács A, Karlsson P *et al*: **Genome-wide multi-omics profiling of the 8p11-p12 amplicon in breast carcinoma**. In*.* Edited by Department of Oncology IoCS, Sahlgrenska Cancer Center, Sahlgrenska Academy at University of Gothenburg, Gothenburg, Sweden; 2017.

14. Krzywinski M, Schein J, Birol I, Connors J, Gascoyne R, Horsman D, Jones SJ, Marra MA: **Circos: an information aesthetic for comparative genomics**. *Genome Res* 2009, **19**(9):1639-1645.
