# Supplementary figures and images for: Clonal relatedness in tumour pairs of breast cancer patients
Source: Breast Cancer Res. 2018 Aug 9;20:96. doi: 10.1186/s13058-018-1022-y (PMC6085699; doi:10.1186/s13058-018-1022-y)

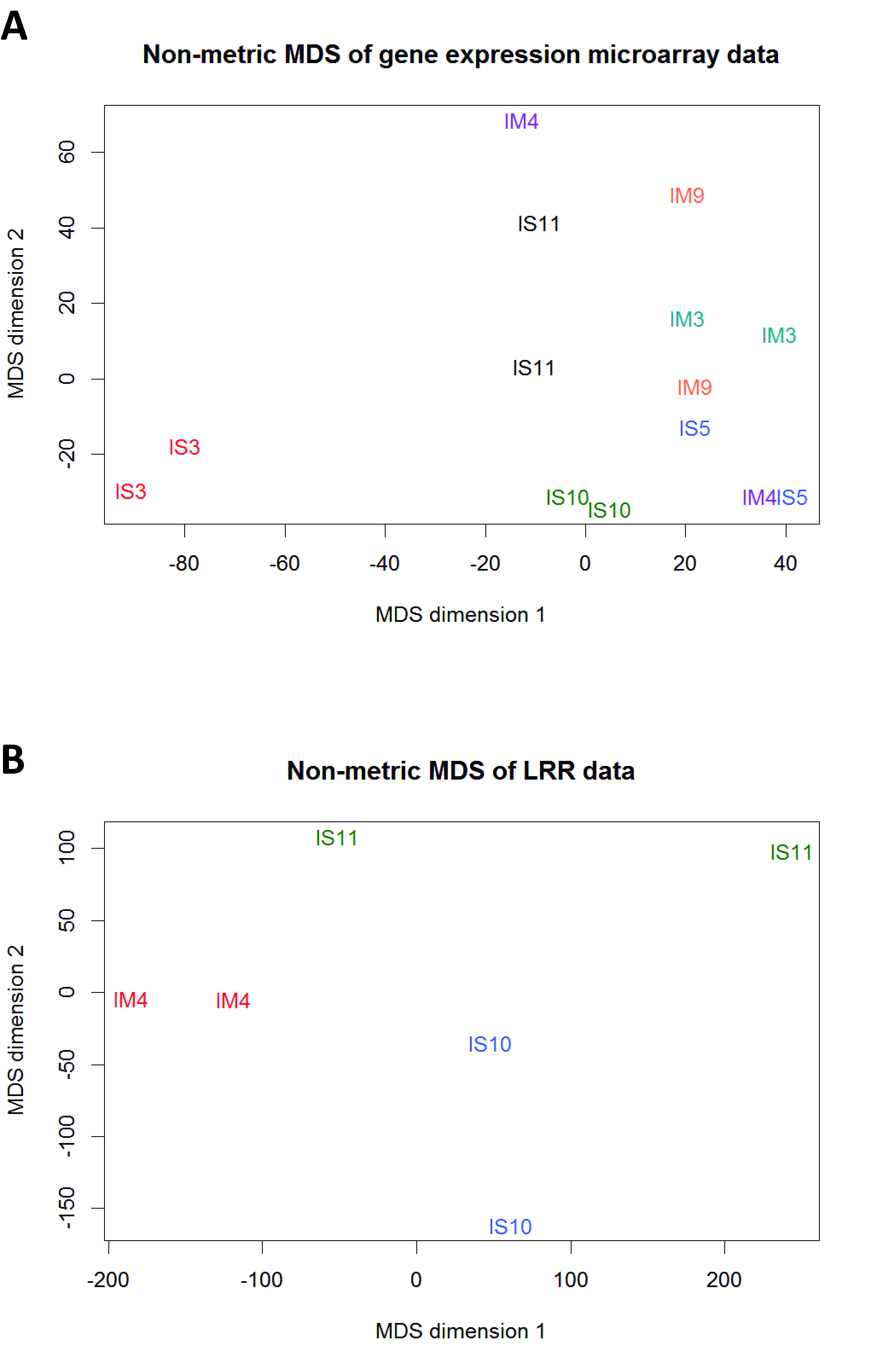

Supplement: Supplementary file 5 — Figure S1. Non-metric multidimensional scaling (MDS) plot of (A) normalised log2 ratios from gene expression data, and (B) LRR values from SNP array data. The MDS plot visualised similarities between the individual samples based on information from the distance matrix. (TIF 1784 kb) [file 13058_2018_1022_MOESM5_ESM.tif]
